# Communities’ perceptions of health hazards induced by climate change in Mount Darwin district, Zimbabwe

**DOI:** 10.4102/jamba.v11i1.748

**Published:** 2019-06-13

**Authors:** Alice Ncube, Margaret Tawodzera

**Affiliations:** 1 Disaster Risk Management Training and Education Centre for Africa, University of the Free State, Bloemfontein, South Africa

**Keywords:** climate change, community perceptions, disaster risk management, health hazard, Mount Darwin, Zimbabwe

## Abstract

Climate change contributes toward many global challenges, such as increases in diseases in some communities, thereby accelerating health hazards to disasters. Establishing the extent to which local communities understand and perceive climate change and related health hazards is important for effective disaster risk management strategies. The objective of this study was to investigate communities’ perceptions of health hazards induced by climate change in Mount Darwin district of Zimbabwe. This was in the light that besides the visible indications that climate is changing, the local people still perceive the climate change phenomenon as mystical or even a non-event. The study was situated within the social capital theory contextualised within the climate change, disaster management and the knowledge and perception realm constructed through social relationships, networks and interactions. A mixed-method research approach was used to assess the extent of knowledge and perceptions related to climate change and climate change-related health hazards. A semi-structured questionnaire was used to survey 204 participants from 10 wards in Mount Darwin, Zimbabwe. Respondents were purposively selected as they were mostly characterised by high vulnerability levels. While 38% of the respondents were not aware of climate change, 7% correctly identified climate change as caused by both natural and man-made forces. Most (89%) of the respondents stated that hazards occur mainly because of meteorological and hydrological causes. The study therefore recommended further education and awareness programmes to deepen community understanding of climate change. Despite the communities having some knowledge gaps and lacking an in-depth understanding of how climate change alters disease, there was some vital information within the Mount Darwin community that could have been used in local disaster risk management initiatives.

## Introduction

Zimbabwe has experienced a warming trend towards the end of the 20th century. Scholars agree that the climate of Zimbabwe is changing although research remains scant (Manyeruke, Hamauswa & Mhandara 2013; Mudzengi et al. 2013; Mugandani et al. 2012). Shifts in rainfall patterns, increases in mean temperature, and increased frequency and extremity in drought, floods and heatwaves have all been cited as evidence of climate change (Peterson et al. 2013). Consistent with global trends, Zimbabwe experienced a warming trend towards the last quarter of the 20th century (Jury 2013). The annual mean temperature has been increasing at 0.4 °C since 1900. The 1990s are ranked the warmest as well as the driest seasons (Chifurira & Chikobvu 2010). During the wet season, day temperatures have warmed compared to night temperatures and this is a cause for concern for the communities.

### Background

Observed and anticipated changes in climate include changes in precipitation, heatwaves, hurricanes and storms. These changes in the surrounding environment affect human health through complex interactions with human behaviour. The changes in climate are often rapid and have widespread threats to human life. Change in climate, either naturally or because of human factors, has certain effects and impacts on agriculture, food security and health of communities. The direct and indirect impacts of climate change are predicted to have severe consequences for African societies and economies (Ajani, Mgbenka & Okeke 2013; Dube & Phiri 2013; Somorin 2010). Rural communities are believed to be particularly vulnerable to climate change (Holmes 2007; Turpie & Visser 2013).Vulnerability of rural households in Africa is caused not only by exposure to climate change, but also by a combination of social, economic and environmental factors that interact with it (David et al. 2007).While literature on climate change has been growing in Zimbabwe (Simba, Chikodzi & Murwendo 2012; Tshuma & Mathuthu 2014), specific locality evidence on climate change, community understanding of the climate phenomenon and evidence on health impacts is still scarce. Establishing the extent to which local communities understand climate change and related health hazards, and their responses, is important for effective disaster risk management.

Research suggests that health is the human dimension that will suffer most the consequences of climate change. The World Health Organization (WHO 2009) identified health hazards emanating from climate change as death from thermal extremes and weather disasters, vector-borne diseases, higher incidence of food-related and waterborne infections, photochemical air pollutants and conflicts driven from depleted natural resources. Earlier, the IPCC (2007) had summed that food, water, industry and settlements will be affected by climate change, consequently worsening the health status of millions of people by increasing deaths, disease and injury because of heatwaves, floods, storms, fires and droughts. The International Federation of the Red Cross (IFRC) (2014) called for more evidence on localised partial perceptions and responses to climate change-induced hazards. It further commented that disaster prevention encounters difficulties where local knowledge, culture and beliefs are unexplored. Similarly, Lotz-Sisitka and Urguhart (2014) reiterated the need for specific and localised knowledge and capacity needs on climate change. Most studies at local levels have concentrated on establishing the impact of climate change, adaptation and mitigation efforts as well as response to climate change impacts, thereby limiting evidence on community understanding of climate change-induced health hazards and their coping strategies (Munaku & Percyslage 2010). This is so despite the signalised importance of community knowledge regarding climate change (Maibach, Nisbet & Weathers 2011).

The study was guided by the social capital theory whose construct has been discussed in the past few decades by authors such as Bourdieu (1977), Coleman (1988), Foley et al. (2001), Gittell and Thompson (2001), James, Schulz and Van Olphen (2001), Putnam (1993, 2000), Booth and Crouter (2001) and Saegert, Thompson and Warren (2001). Knowledge, perceptions and various capacities of the communities are influenced by their social capital. Households are made up of networks and these networks are not passive; they possess social assets acquired, developed, improved and transferred across generations and societies. Social networks are built upon values, norms, knowledge, social learning and information sharing that is vital in the climate change adaptation and perceptions discourse. Communities that have effective networks based on mutuality and reciprocity are better able to share information and transfer knowledge. This influences the extent of common information among the public regarding climate change and related hazards. Through informal networks, households share information on hazards and disasters, factors leading to these forces and the actions at individual or collective levels to address and reduce exposure and vulnerability to disasters related to climate change. Culture as a component of social capital influences rights, worldviews, world perceptions, identity and access to knowledge, governmental relations and resource availability. Islam (2013) argued for indigenous knowledge as a form of social capital. This knowledge evolves from the different sources within the social process, built through associations of groups, accumulated through close contact with nature and evolves in the local environment in a creative and experimental manner. Indigenous knowledge systems (IKS) are dynamic, constantly incorporating outside influences and inside innovations and have been tested and found valid in the local context (Johnson 1992; Nhemachena et al. 2014).

Climate change and related hazards information can therefore be exchanged through social networks. As with building resilience of communities against socio-economic vulnerability, social capital is important in building the resilience of communities against climate change hazards. Social capital is important in the climate change and subsequently induced health hazards discourse. Through social capital, climate knowledge is created and shared, coping strategies are determined and the propensity and priority to adapt to climate change health hazards are influenced (Smit et al. 2000). Ellen and Harris (1996) underscored the importance of social capital such as the skills, experiences and insights of people that are applied to maintain or improve livelihoods and can be incorporated into the development process of agriculture, use and management of natural resources, healthcare, community development and poverty alleviation, among others.

### Conceptualisation of climate change-induced health hazards

Health hazards because of climate change are diverse and complex. Health hazards have been defined as potential consequences of reduced state of physical, mental and social well-being of the population (IPCC 2012). These include heat stress, vector-borne diseases (such as malaria, dengue and yellow fevers), extreme weather events, air pollution, communicable diseases (such as cholera) and non-communicable diseases (such as cardiovascular and respiratory diseases) (Muzhedzi & Cele 2014). The deleterious impacts of climate change are further expected on mental and occupational health, food insecurity, hunger and malnutrition. Wardekker (2011) argued that hazards because of climate change would have health effects that include an increased burden of malnutrition, diarrhoeal, cardiorespiratory and infectious diseases, increased morbidity and mortality from heatwaves, floods and droughts, changes in distribution of some disease vectors and hence substantial burden on the health delivery system.

### Respondents’ perceptions of health hazards induced by climate change

Empirical examinations on the knowledge and perceptions about climate change-related hazards and response strategies are not new although they are very few. According to Rusinga et al. (2014), studies on knowledge and perceptions of climate change and related hazards underline the following:

- Communities have developed systems that adapt to local, natural and environmental conditions and manipulate micro-climate.
- Sustainability of interventions to reduce the hazards of climate change depends on people’s knowledge and ability to adapt.
- Community decisions are related to information acquired through records or interacting with natural environment.
- People have always been experimenting through interaction with weather and climate.
- Responses to climate change would be sustainable when people’s knowledge, values and priorities are recognised.
- It is necessary to understand the way people see and understand their vulnerability and needs.
- Indigenous knowledge is intuitive and experimental.

Abdel-Monem (2014) argued that studies on the knowledge and perceptions of climate change and responses have been undertaken as ways to measure and track climate change effects and develop effective and sustainable interventions. Social scientists are able to assess the changing environment of people’s knowledge towards climate and climate-related hazards and disasters. In the 1990s, these studies were anchored on establishing public awareness on climate change, while early efforts were meant to gauge public understanding of climate change causes and effects (Bostrom et al. 1994; Read et al. 1994) together with the associated value judgements (Kimpton et al. 1996). In other contexts, studies were to establish respondent characteristics, and associated perceptions and attitudes towards climate change. The relationship had been found to be complex and unclear.

Local people’s perceptions, impacts and adaptation studies are initiated because there is limited scientific evidence to alert policy-makers to the hazards of climate change at local level (Bhusal 2009). Scientific evidence is locally narrow and globally wide, leaving little information known about community-learned experiences on natural hazards. At the core is the recognition that vulnerability exists today, it will not disappear on its own, but is growing and there is a need to make active interventions that can reduce the extent to which communities are vulnerable. While scientific evidence has grown, credible evidence on the knowledge of practical community experiences with climate change hazards, impacts and responses needs to be exploited. This body of research and evidence is more important as the natural hazards on climate change have worsened, impacting negatively agriculture, health, food security, hunger and life experiences (Bhusal 2009; Mata-Lima et al. 2013).

This social knowledge is time-, place- and culture-specific and hence cannot be easily generalised. It needs to be updated continuously through research, as it is both dynamic and innovative. The local knowledge is used to respond to hazards such as droughts, floods, storms, famines and extreme events. It has been found to match quantitative climate data in some settings while diverging from the same in other settings (Ogalleh et al. 2012).

The scientifically identified, described and measured climate change impacts and vulnerability may differ from household perspectives. Leiserowitz et al. (2010) and Dessai, Kandlikar and Risbey (2005) stated that lay public perceptions’ and interpretation of climate change and its hazards are based on psychological, social, moral, institutional and cultural processes. The expert definitions based on probabilities and severity of consequences are narrower than the lay public, which have multidimensional and complex sets of assessments. Public perceptions regarding climate change and hazards of climate change are not only shaped by scientific and technical descriptions of hazards, but by a variety of psychological and social factors that encompass personal experience, effect and emotional, imagery, trust, values and worldviews (Slovic 2012).

## Research methods and design

### The study area

**FIGURE 1:**
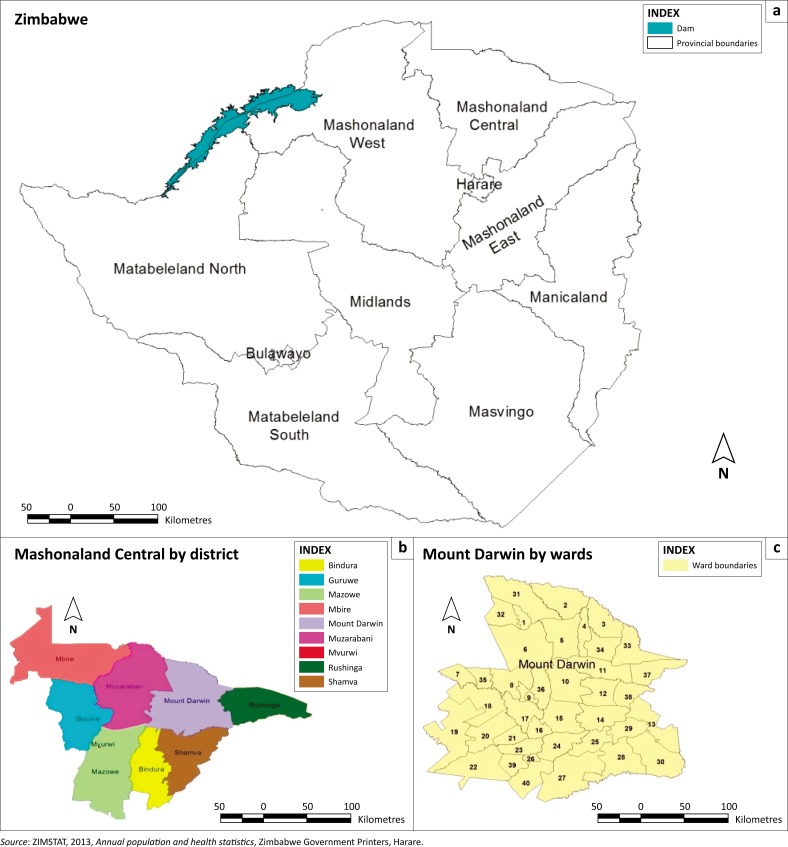
Maps for Zimbabwe, Mashonaland Central province and Mount Darwin district.

The study was undertaken in predominantly rural Mount Darwin district in Mashonaland Central province of Zimbabwe (see Figure 1). The district had an estimated population of 218 724 out of a total provincial population of 1.088 million (ZIMSTAT 2013). It is the most populous district among the eight districts, accounting for 20% of the provincial population. The average temperature in the district is 24 °C in summer and 14 °C in winter. The average annual rainfall is between 650 mm and 800 mm and falls in the summer season (mainly between November and February). The district is predominantly between agro-ecological regions 4 and 5.

### Materials and methods

The mixed-method research approach was utilised in this study. A semi-structured questionnaire consisting of qualitative and quantitative questions was designed (Finn & Jacobson 2008; Kothari 2004). Data were gathered through purposive random sampling technique, targeting older respondents who were likely to have been exposed to different climatic events for a longer period in the area. Ten out of 40 wards in Mount Darwin district were identified using a stratified purposeful sampling technique (Patton 2002). Using the Government of Zimbabwe central statistics mapping frame, the 10 wards were 1, 2, 3, 4, 5, 6, 11, 16, 19 and 23 (ZIMSTAT 2013). These wards were the most populous and rural wards in the district, representing 29% of the district population. The population in these wards was homogenous, being prone to climate-induced hazards on a perennial basis and with high poverty levels, thereby high morbidity rates (Jager, Putnick & Bornstein 2017). A sample of 250 households was used based on the homogenous convenience tactic, although 204 respondents completed the questionnaires fully. Permission to access the participants was given by the Zimbabwe Ministry of Health and Child Care at the Mount Darwin district health office. The local traditional leadership was also consulted and advised about the presence of the researchers. The participants were also apprised of their rights to participate in the survey and that they would remain anonymous. The ethics clearance letter from the university was used to introduce the researchers.

### Data analysis

The respondents’ cross-sectional information was both qualitative and quantitative. The qualitative information yielded non-numeric responses and arbitrary categorises that were coded into themes, and the quantitative numerical responses were meaningfully manipulated using conventional arithmetic. Results were presented to reflect on the knowledge of climate change, perceptions about climate status, hazard occurrences, health effects of climate change, the occurrence of diseases attributable to climate change, actions to reduce climate change-induced health hazards and actions being undertaken by respondents to reduce the health hazards posed by climate change. The respondents’ perceptions of the current climate status as well as climate change at the time of their settlement in Mount Darwin were measured on a five-point Likert scale ranging from extremely bad to very good. Unstructured or qualitative responses synthesised by examining the recurring responses or a list of responses from the respondents are summarised in boxes.

### Ethical considerations

Ethical clearance was obtained from the University of the Free State (tracking number: FS-HSD2016/0586).

## Results and discussion

Of the study population, 204 respondents (75% men and 25% women) fully completed and returned the questionnaire (see Table 1). Of the respondents, 43% indicated that they possessed secondary education and 8% indicated to have a higher education. Multiple livelihood options were mentioned by 90% of respondents and 88% mentioned of food cropping.

**TABLE 1:** Demographic, knowledge and perception responses of respondents.

Variable	Category	Number of respondents	%
Gender	Male	152	75
	Female	52	25
Marital status	Single	7	3
	Married	154	76
	Divorced	17	8
	Widowed	26	13
Education level	Standard 4	45	22
	Standard 6	31	15
	Primary	25	12
	Secondary	87	43
	Higher	16	8
Livelihood (multiple responses)	Livestock production	184	90
	Food cropping	180	88
	Cash cropping	161	79
	Vegetable production	106	52
	Remittances	106	52
	Others (petty trade, hunting, casual labour, etc.)	41	20

### Respondents’ knowledge and perceptions of climate change

The respondents were asked if they had ever heard of climate change and the causes of climate change, the results of which are given in Table 2. One hundred twenty-six respondents indicated that they had heard about climate change. Of the 126 who had heard about climate change, 42% indicated that climate change is caused by man-made phenomenon, while 37% considered it a natural phenomenon, 14% referred to climate change as caused by spiritual forces and 7% indicated that both man-made and natural forces cause climate change. The respondents were asked about their perceptions regarding the current climate status as well as the perception of climate change at the time of their settlement in Mount Darwin (see Table 2). On the perception of the current climate status, 36% and 39% regarded climate as extremely bad and very bad, respectively.

**TABLE 2:** Respondents’ knowledge and perceptions of the current climate status as well as the climate change at the time of their settlement in Mount Darwin.

Climate change	Respondents’ knowledge and perceptions of climate change	Respondents’ response	Number of respondents	%
Knowledge	Have you ever heard of climate change? (*n* = 204)	Yes	126	62
		No	78	38
	What are the causes of climate change? (*n* = 126)	Man	53	42
		Natural	47	37
		Spiritual	18	14
		Both (man and natural)	8	7
Perception	Current variation of climate (*n* = 204)	Extremely bad	73	36
		Very bad	80	39
		Bad	51	25
	Climate variation at the time of settlement (*n* = 204)	Extremely bad	3	2
		Very bad	15	7
		Bad	38	19
		Good	117	57
		Very good	31	15

There was a misconception among the respondents that climate change was caused by spiritual forces. This result matches because of the earlier study carried out in Bangladesh where 46% of the respondents opined that climate change is a phenomenon caused by nature or God (Haines et al. 2006). Furthermore, the finding was consistent with findings in Nigeria where the majority of respondents identified man-made forces as responsible for climate change compared to natural forces (Nwankwoala 2015).

The respondents were required to explain what they understood about climate change. Box 1 indicates the respondents’ understanding of the meaning of climate change. Among those who had heard about climate change (*n* = 126) interpreted it as changes in weather or components, such as rainfall, wind, heat and temperatures as well as sudden changes in the expected and usual weather. Generally, respondents interpreted climate change as simply changes in weather components like rainfall and temperature was recurring more often. The results augur well with studies that found households interpreted climate change as changes in rainfall, temperature, storms, floods, deep cold and long heatwaves (Ogalleh et al. 2012; Toan et al. 2014). The respondents also highlighted the intensity and predictability of rains and wind as indications of the changing climate. Conflicting views and interpretations of climate change showed the respondents’ poor understanding of the climate change phenomenon, which was similar to the results of Abdel-Monem (2014). Climate change was understood as change in weather such as rainfall and temperature fluctuations. This is similar to findings by the Asian Foundation in Bangladesh. However, this study found that respondents related climate change to weather phenomenon becoming unusual. In Bangladesh, the Asian Foundation found that climate change was linked to strong climatic events normally experienced such as floods, droughts, heavy rain, storm or cyclone and high temperatures. Although not frequent, some respondents in this study mentioned heavy rains, droughts, excessive heat and cyclones as outcomes of shifting weather conditions because of climate change. The major departure from related findings is that in those settings in the current study, climate change was largely viewed as strong climatic events, which were likely to result in disasters.

Box 1 Respondents’ understanding of the meaning of climate change.

- Change of weather such as rainfall and fluctuations in temperatures
- Change in weather of a place
- Change in weather arrangement, focus and erratic rains
- Change of rainfall dates, weather and low rainfall nowadays compared to the 1980s when we received rainfall between 400 mm and 900 mm every year
- Changes in rainfall, temperature, humidity and many other factors
- It means changes in temperature, wind, rainfall, amount of heat and so on.
- Natural change of seasons, maybe because of elements of weather like high or low and unreliable rainfall during summer season
- Sudden change of the expected and usual weather conditions
- The unusual presentation of seasons in a year
- Change of weather and conditions
- The unusual presentation of seasons in a year, year after year, some of which are not favourable to human life
- It is a change in weather patterns from the usual climatic conditions
- Change of weather–rainfall patterns over time
- The change in the weather and all that happen that affects rainfall and agriculture
- It means the change in weather in our area such as rainy season
- Changes of weather patterns
- Climate change means seasonal weather such as rainfall dates, hot season temperature change
- It refers to changes in rainfall patterns, temperature, heat and wind, among others
- Change of seasons (*kuchinja kwemwaka*)
- It is a shift in weather conditions from those we used to experience to heavy rains, droughts, excessive heat and cyclones
- Change of the seasons such as the months when certain weather phenomenon used to take place. Maybe change in intensity of weather elements, for example, rainfall, rainfall intensity
- Change in the direction rains come from.

### Perceptions of climate change trends

Respondents had to indicate their perceptions of trends of key climatic status (Box 1, Table 3). About 169 (83%) respondents indicated that rainfall had decreased, 17 (8%) stated that it has remained constant, while 18 (9%) reported they were not sure. At least 165 (81%) of the 204 respondents felt that temperature, drought frequency, dry spells and heatwaves had increased. About 101–161 (50% – 79%) respondents stated that wind, sun heat and floods had increased, while merely one to three respondents were of the opinion that it had decreased. The rest were not sure or were of the opinion that it was constant.

**TABLE 3:** Respondents’ perceptions of rainfall and climate trends.

Variable	Decreased		Constant		Not sure		Increased	
	*N*	%	*N*	%	*N*	%	*N*	%
Rainfall	169	83	17	8	18	9	-	-
Temperatures	1	1	12	6	17	8	174	85
Wind	-	-	38	19	46	23	120	59
Sun heat	1	1	16	8	26	13	161	79
Drought frequency	8	4	8	4	-	-	188	92
Floods	3	2	44	22	56	28	101	50
Dry spells	-	-	13	6	18	9	173	85
Heatwaves	-	-	9	4	30	15	165	81

To validate and evaluate consistency in responses, the respondents were asked to indicate on the major observed shifts in rainfall and temperature (Box 2). The respondents indicated that deceasing rainfall, shorter rain seasons, increased temperatures, recurring heatwaves, increasing hot days, increased hot and cold weather and abnormal unusual temperatures are the observed shifts.

Box 2 Observed major abnormal shifts in rainfall and temperature patterns.

- The amount of rainfall has decreased over time; temperature is increasing especially in October.
- Rainfall has decreased; temperature has gone high.
- Used to receive rainfall between 400 mm and 900 mm, that of region 4. Now it has decreased to between 150 mm and 300 mm, and in the 1980s–1990s, temperatures were constant but now we have recurring heatwaves.
- Rainfall season has shortened, at times only 2 months or less. Excessive heat, heat that leads to deaths of birds and wild animals.
- It is difficult to plan as rainfall can come for only 3 months; Mount Darwin is having high temperatures that do not support agriculture.
- Rainfall is erratic and has resulted in water shortages in the area; temperatures are slightly hot nowadays but not very hot.
- Rainfall is now erratic; high temperatures are now being experienced with heatwaves currently recurring.
- In earlier days, we used to receive rainfall in mid-November up to May, but now we are receiving rains late December. We are having high temperatures throughout the year now, though earlier temperatures were high during mid-September to November.
- Rainfall has decreased by far.
- We used to have rains from mid-November to mid-May but now we receive it in late December to early February. The temperatures were high in mid-September to mid-November, but now it is hot all year-round.
- Decrease in the amount of rainfall and type of rainfall. Temperature is no longer constant – sometimes very cold and at other times too hot, experiencing heatwaves.
- Rainfall has decreased in amount as compared to earlier years such as 1997. High temperatures are experienced throughout the year.
- Rainfall is low and temperature has increased.
- Rainfall no longer comes normally since the 1990s.
- Rainfall has decreased; temperature has been increasing because of low rainfall.
- Rainfall during 1999–2001 was very good but since 2002 there has been very short rainfall season.
- No rains in 2015; temperatures moderated in 2015.
- The season has changed drastically; earlier we used to receive rains for 3 months but now only for a month or a month and a half. Temperatures have risen to unsatisfactory levels – too much heat affecting human habitation.
- More extreme rain events, temperatures frequently exceeding the 400°C, which were never recorded in the past.
- The rainy season has become short; the area receives a heavy rain that destroys both homes and crops. Temperatures are increasing, causing discomfort among people.
- The temperatures are now consistently hot.

The respondents’ views regarding the current climate status augured well with the existing body of literature on climate change in Zimbabwe. The current climate was not favourable to the respondents as shown by the overwhelming rating, ranging mostly between very bad to extremely bad as compared to the time at settlements. The respondents viewed rainfall as decreasing, while temperatures were increasing together with wind speed, sun heat, drought frequency, floods and dry spells. This was consistent with findings in the literature (Nhemachena et al. 2014; Simba et al. 2012). Simba et al. (2012) argued that in the study conducted in Masvingo province of Zimbabwe, respondents regarded climate is changing because of the increasing frequency of droughts, dry spells, shifts in rain seasons, rising temperatures, declining rainfall amounts and more frequent mid-season droughts. Households’ views might have been shaped by their interaction with the environment over the years. The correct interpretation of the respondents’ knowledge regarding the environment raised hope in raising the awareness of the community about the health hazards posed by climate change.

### Respondents’ perceptions of climate change effect on their health

The respondents were asked whether they thought that climate change affected their health and whether it had anything to do with the health hazards being experienced in Mount Darwin because of changes in rainfall, cold, flooding and heat. A total of 134 (66%) respondents stated that climate change affected their health, while 70 (34%) told that it did not affect them. As shown in Box 3, most (99%) of the respondents thought malaria as a health hazard emanating from climate change, while only 51% considered malnutrition as a reason for the same.

Box 3 Respondents’ perceptions of how climate change impact their health (*n* = 204).

- Too much rainfall brings about floods, and hence outbreaks of diseases, hunger and malnutrition.
- Rainfall has become erratic and food shortages occur, with diseases rising.
- Lack of food for the family, loss of weight.
- Unable to feed my family because I depend on farming as an activity, thus putting my family at risk of poverty.
- Unable to feed my family because I depend on agriculture to survive.
- It affects agriculture because crops would not grow well and animals die because of starvation and hunger as a result of shortages of water caused by low rainfall. As a result of high temperature, crops wilt and animals die. Death of livestock because of lack of water and pasture resulting in inadequate access to protein in diet.
- Climate change can cause scarcity in water resources, thereby causing diarrhoea. It can lead to vector breeding. Climate change is the major cause of most health problems we are experiencing in the community.
- Outbreaks of diseases which affect people and livestock.
- During hot weather, we do not sleep in mosquito nets.
- Livestock sold unplanned and at unprofitable prices. Disease outbreaks and increase. People who are poor because of climate change are dying without seeking proper medication at better health centres because they do not have enough money from either their livestock or their cash crops.
- Temperatures go beyond levels that our bodies are used to and new infections are emerging.
- We will not have enough food and hence malnutrition.
- Because of the changes in climate, many people are dying.
- Sun heat causes headache in both adults and children.
- When temperatures increase, mosquitoes breed fast, resulting in enhanced spread of malaria, headache and dysentery.
- New diseases emerge while people frequently fell sick (*hosha dzisakamboonekwa dzinotanga uye vanhu vanongorwararwara*).
- Lack of rainfall is causing most of the diseases; people are drinking water from the same sources as animals, which causes many diseases.
- Climate change results in shortage of proper diet and unavailability of water for activities such as gardening and feeding of domestic animals.
- High temperatures cause skin damages and excessive sweating during the day and at night, leading to dehydration and body weaknesses.
- Too little and too much rainfall cause food scarcity, too much temperatures such as heat waves exhaust the body and mosquitoes become active and hence malaria becomes a problem.

**FIGURE 2:**
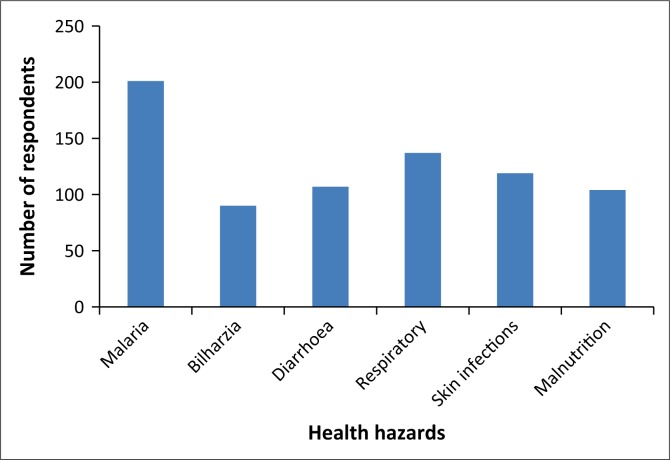
Respondents’ perceptions of the health hazards experienced because of changes in climate status (*n* = 204).

The respondents were asked to explain how climate change affected their health. Only 73 respondents stated that climate change affected their health (see Figure 2). Most of the respondents identified the effect of climate change on health as the increasing scarcity of food that results in malnutrition and related diseases. Droughts and excessive rains resulted in hunger and malnutrition. Water shortages resulted in outbreaks of diseases such as cholera and diarrhoea. In addition, climate change resulted in deaths and unplanned sale of livestock, fetching low prices, that affected people’s income and hence access to health services and nutrition. Excessive rains and temperatures caused new and emerging diseases that were not common in the area. High temperatures also caused headache, dysentery, diarrhoea, skin damages and excess body sweating, leading to dehydration and weaknesses; mosquitoes were also mentioned as a cause that spread malaria. The respondents therefore viewed the health effects of climate change in varied ways; however, a greater proportion of respondents were not able to articulate these health effects.

The study also sought to examine how climate change has altered disease manifestation among the community of Mount Darwin. It was clear that the respondents found it difficult to express how climate change altered disease prevalence in their community. The knowledge gap was observed in this instance whereby respondents failed to articulate correctly how climate change increased disease manifestation as presented in Table 4.

**TABLE 4:** Respondents’ perceptions of how climate change has altered the disease manifestation (*n* = 204).

Disease manifestation
**Malaria** Activated because mosquitoes which spread malaria favour hot areas Has increased because of favourable breeding sites High temperatures Less rainfall results in less mosquito, hence less cases of malaria Changes in temperatures and rainfall patterns affect the breeding of mosquitoes, which affect the transmission of malaria
**Bilharzia** Unclean water Increase in heat Drinking of unsafe and unclean water from dams sharing with cattle
**Diarrhoea** Occurrence has increased because of poor water availability Low rainfall, water and sanitation Drinking of unsafe water because of water shortages Little or no water Drought Little and dirty water Dirty water Poor diet Sharing water sources with animals
**Skin infections** Because of excessive heat Heatwave Excessive sun heat Cross infections Change of weather
**Malnutrition** Because of droughts, poor rainy seasons, poor distribution of rain days, shorter rain periods and changing suitable crops Lack of food Imbalanced diets because of droughts
**Respiratory infections** Experienced because of excessive winds and dust, too much cold, extreme cold days Heat increasing Blowing wind Cold Unpredictable and constantly changing temperatures

The results in Table 4 are mixed. In some cases, the respondents were able to articulate the channels through which disease prevalence was altered by climate change. In the case of malaria, the respondents indicated that changes in both rainfall and temperatures affected the breeding of mosquitoes, which spread malaria. Some respondents thought that bilharzia was altered by shortages in water, which forced people to share water sources with animals. The increase in diarrhoea in the community was clearly understood by the respondents, who stated that people were forced to drink dirty water from unprotected sources because of extreme drought. It was, however, not easy for the respondents to articulate how temperature rise and heatwaves caused skin diseases and respiratory infections.

Hazards were pervasive; however, those who experienced these hazards were not aware of the link between these hazards and climate change. Most of the respondents (89%) stated that they had encountered some form of meteorological and hydrological hazards. However, contrary to expectations and findings from other studies (Lujala, Lein & Rød 2014; Menny et al. 2011), climate change knowledge about repository had been found to be unrelated to personal experience with these hazards. Lujala et al. (2014) stated that climate change knowledge, attitudes and perceptions are explained by direct personal experience of damage posed by climate-related events such as floods and landslides. Related to the argument that personal experiences of hazards pose a greater impact on climate knowledge, Menny et al. (2011) argued that experience of extreme weather events affects the knowledge regarding climate change. The finding therefore implies a huge climate change knowledge deficit even among those who had experienced climate change-related hazards.

Some respondents were, however, knowledgeable about climate change-induced hazards. The few respondents who managed to answer the question on climate change-induced hazards agreed that altering hazard patterns exposed communities to vulnerability. Climate change factors were linked to cyclones, severe water shortages, extreme weather events and drought, among others. Respondents mentioned other factors such as poverty, migration, overcrowding as well as poor facilities, infrastructure and planning that exacerbated vulnerability to hazards.

Two-thirds (66%) of the respondents regarded climate change as an intervening variable for their health. The respondents opined that climate change altered diseases such as malaria, respiratory infections and skin infections, while fewer respondents agreed on the same for bilharzia, diarrhoea and malnutrition. Similar results were posited by the Caribbean Institute of Media and Communication (CIMC) (2012) when about 63% of the respondents stated that climate change was related to health epidemics. The Asian Foundation (2012) found 80% of the respondents confirmed that health hazards were among the household-level impacts of climate change. Thus, the findings of this study are within the confines of related studies. The health impacts of climate change mentioned tally well with some literature (IPCC 2012; Menny et al. 2011; The Asian Foundation 2012).

Heat-related mortality among the elderly, chronically sick, very young and those socially isolated were identified by the IPCC (2012) as some of the health hazards posed by climate change. Menny et al. (2011) added changes in infectious diseases, scarcity of drinking water, increased malnutrition, increased death, diseases and injury as emanating from extreme climatic events. Other health hazards as espoused by Menny et al. (2011) include increased burden of diarrhoeal diseases, increased frequency of respiratory diseases and increased spatial distribution of some infectious diseases. Menny et al. (2011) further found that 25%, 37%, 38% and 30% of the respondents in their study regarded health hazards as emanating from drought, flood, flash floods and cyclones, respectively. While the general effects of climate change on health were identified in this study, an in-depth understanding regarding the impacts of these climatic events on different societal groups (such as the young and the elderly and the rich and the poor) was deficient

Nhemachena et al. (2014) and Moyo et al. (2012) found that the effects of climate change were agriculture-biased although about 34% of the households regard climate change as having an impact on health. The respondents mentioned that in 2011 some people died in Hwange because of heat stress. In addition, Nhemachena et al. (2014) stressed that the general perceptions about the impacts of climate change are that it adds to other multiple factors such as poverty, HIV and AIDS and food insecurity to worsen health outcomes. Haines et al. (2006) identified similar hazards of climate change, namely, altering of microbial proliferation causing food poisoning, unsafe drinking water, changes in vector–pathogen–host relations and infectious diseases, as well as geographical changes in the occurrence of malaria, dengue and viral diseases. Changes in mean climatic conditions and variability alter the ecosystem, manipulating crops, livestock and other yields with a nutritional effect on households, while also causing environmental degradation. In addition, Haines et al. (2006) noted that environmental degradation would result in displacements that will worsen poverty and exacerbate health effects such as mental health, infectious diseases, malnutrition and physical injuries. Thus, the study results on the health hazards posed by climate change were within the parameters of existing literature.

However, majority of households generally lacked the knowledge regarding the exact mechanisms through which health was affected by climate change. These findings are similar to other studies as the majority of the respondents were not knowledgeable about the exact channels through which health outcomes were altered by climate change. The South African medical Journal (Myers et al. 2011) stated that even though the people’s perceptions of health hazards of climate change tally with well-documented evidence, they lack knowledge regarding the exact ways through which various impacts of climate change are transmitted.

## Conclusion and recommendations

In this study, we examined the knowledge of and responses to climate change-induced health hazards in Mount Darwin district, Mashonaland Central province, in Zimbabwe. The study emphasised on valuing community-level knowledge of climate change, hazards and responses; in particular, it emphasised understanding local knowledge that is important in shaping effective policies and strategies for reducing exposure and vulnerability and building resilience to the health hazards of climate change.

A fairly high proportion (38%) of the respondents were not aware of climate change. The respondents were not aware of the causes of climate change as only 7% understood that climate change is both a man-made and natural phenomenon. Furthermore, about 14% of respondents believed that changes in climate were related to spiritual forces. Knowledge about climate change was interpreted as changes in rainfall amount, pattern and intensity, changes in temperatures, frequency and intensity of drought, other sudden changes in expected weather patterns and unexpected weather events. The definitions were more consistent with the scientific interpretations of climate change despite some variations. Unfortunately, only a few respondents mentioned these qualitative descriptions of climate change.

The respondents generally supported that hazards were occurring in Mount Darwin. Meteorological and hydrological hazards were more common, followed by biological hazards and geophysical hazards. As majority of respondents further agreed that the occurrence patterns of hazards were changing, they were not able to qualitatively explain the factors causing changes in hazard occurrence, especially the role of climate change.

Households perceived current climate as bad or extremely bad characterised by decreasing rainfall amount, increasing temperatures and frequent dry spells, drought and heatwaves. Furthermore, the respondents agreed that the occurrence of diseases attributable to climate change had increased, and it was now high or very high in both summer and winter compared to the past 5–10 years. While the majority of respondents stated that changes in climate affected their health, the proportion of those who felt otherwise was low (44%). The study also found that respondents were not able to articulate the channels through which specific health hazards, such as skin infections, were altered by climate change. On the positive side, the households were able to indicate that climatic variables such as temperature could have multi-level health hazards.

Drawing on the established available knowledge, actions against hazards of climate change and the knowledge gaps that exist in the community of Mount Darwin, the following policy recommendations are suggested. There is a need for households to have the correct information relating to the causes of climate change and subsequent health hazards in order to demystify the relationship of climatic change issues with spiritual forces. Hence, climate change education is necessary to broaden and build the knowledge of households and bring convergence among them regarding understanding climate change. It is necessary for the relevant ministries. The newly created Zimbabwe’s National Climate Change Response Strategy should be implemented in order to build adaptive capacity in communities, such as Mount Darwin, that are affected by climate change. As 43% of respondents in the study stated that they had secondary education, the informal education programmes for adults could be designed to assist the community further in the climate change phenomenon. Public awareness programmes on the relationship between extreme weather conditions (such as extreme cold) and chronic illnesses (such as asthma and chest pains) are necessary in order for the community to mitigate and prevent exposure to such conditions. These awareness programmes need to utilise the existing knowledge and new knowledge in order to get the community’s approval and involvement.
